# Coronary artery calcifications are not associated with epicardial adipose tissue volume and attenuation on computed tomography in 1,945 individuals with various degrees of glucose disorders

**DOI:** 10.1016/j.ijcha.2025.101613

**Published:** 2025-01-20

**Authors:** David Molnar, Elias Björnson, Ola Hjelmgren, Martin Adiels, Fredrik Bäckhed, Göran Bergström

**Affiliations:** a Department of Molecular and Clinical Medicine, Institute of Medicine, Sahlgrenska Academy, University of Gothenburg, Gothenburg, Sweden; b Department of Radiology, Sahlgrenska University Hospital, Region Västra Götaland, Gothenburg, Sweden; c Department of Clinical Physiology, Sahlgrenska University Hospital, Region Västra Götaland, Gothenburg, Sweden; d Pediatric Heart Centre, Queen Silvia Childreńs Hospital, Sahlgrenska University Hospital, Region Västra Götaland, Gothenburg, Sweden

**Keywords:** Epicardial adipose tissue volume, Epicardal adipose tissue attenuation, Non-contrast computed tomography, Coronary artery calcium score, Asymptomatic population, Pre-diabetes

## Abstract

**Background:**

The quantification of coronary artery calcifications (CAC) is a mainstay in radiological assessment of coronary atherosclerosis and cardiovascular risk, but reflect advanced, possibly late-stage changes in the arteries. Increased volume and changes in attenuation of the epicardial adipose tissue (EAT) on computed tomography (CT) have been linked to adverse cardiovascular events, and these changes in the EAT might reflect earlier stages of the processes leading to clinically manifest atherosclerosis. The relationship between EAT and CAC is subject to a knowledge gap, especially in individuals with no previously known coronary artery disease.

**Methods:**

Fully automated EAT analysis with an artificial intelligence-based model was performed in a population sample enriched for pre-diabetics, comprising a total of 1,945 individuals aged 50–64 years, where non-contrast CT images, anthropometric and laboratory data was available on established cardiovascular risk factors. Uni- and multivariable linear regression, gradient-boosting and correlation analyses were performed to determine the explanatory value of EAT volume and attenuation data with regards to CAC data.

**Results:**

Neither EAT volume nor EAT attenuation was associated with the presence or severity of CAC, when adjusting for established cardiovascular risk factors, and had only weak explanatory value in gradient-boosting and correlation analyses. Age was the strongest predictor of CAC in both sexes.

**Conclusion:**

No independent association was found between CAC and total EAT volume or attenuation. Importantly, these findings do not rule out early stage or local effects on coronary atherosclerosis from the EAT immediately surrounding the coronary arteries.

## 1 Introduction

The presence of calcifications in the coronary arteries is one of the most specific signs of coronary atherosclerosis[1], [2], [3]. They can be detected and quantified reliably with computed tomography (CT), and the Coronary Artery Calcium Score (CACS) remains an important radiological assessment tool for the evaluation of the extent of coronary atherosclerosis[4], and the risk of future cardiac events[5]. Several interrelated risk factors are relevant to the pathogenesis of coronary atherosclerosis, such as age, sex, smoking, hypertension, hyperlipidemia, glucose disorders and obesity[1], [6], [7], [8]. In recent decades research on the epicardial adipose tissue (EAT), which immediately surrounds the heart muscle and envelops the coronary arteries, has gained much traction. It has been shown that the EAT exhibits increased inflammatory cytokine activity in coronary artery disease[9], [10], and with the advent of technological improvements in the field of CT, both the EAT volume (EATV) and EAT attenuation (EATA) have been shown to be associated with various aspects of coronary atherosclerosis[11], [12], [13], [14], [15], [16]. Much of the findings in the literature postulating an association between EATV or EATA and coronary artery atherosclerosis have relied on either small cohorts, selected populations with oversampling of diseased individuals[13], [17], or analysis of only partial volumes of EAT. Relatively scarce, and to some extent contradictory, data is available on the EAT and coronary calcifications in a population more representative of the general population, and on the relative strength of the association in comparison to other established anthropometric, metabolic and social risk factors[15], [18]. One reason might be that until recently only cumbersome and time-consuming manual or semi-automatic methods have been available for the analysis of EAT[19]. We have previously developed a software model capable of fully automated analysis of both EATV and EATA data in large cohorts with a precision and accuracy comparable to human expert measurements, and a failure rate of between 0.5 and 1 % requiring post-analysis manual intervention[20]. The aim of the present study was to test if there is an association between CACS and EATV or EATA in a population sample enriched for pre-diabetic and diabetic individuals. Four different statistical models were applied to characterize the relationship between CACS and EATV/EATA and relevant co-variates and confounders.

## 2 Methods

### 2.1 Population

Data from the “Microbiota, development of type 2 diabetes and cardiovascular disease study” (IGT-study) was used. A total of 1,965 subjects were included in the IGT-study, and from these a total of 1,945 individuals had retrievable CT images for CACS- and EAT analyses and could be included in the current study.

Selection of participants has been described in detail elsewhere [21]. Briefly, men and women 50 to 64 years of age born in Sweden were randomly selected from the Swedish population registry and invited to a screening visit. A fasting capillary glucose and an oral glucose tolerance test (OGTT) conforming to the WHO criteria[22], [23] were performed, and individuals were eligible for inclusion if results reflected type-2 diabetes (T2D), pre-diabetic states, or, irrespective of test results, had an increased assessed risk of developing T2D (FINDRISC, score > 14 [24], or 2 first degree relatives with T2DM). Individuals with normal tests and no increased risk of developing T2DM were randomized (1:4) to inclusion. Exclusion criteria were: known diabetes, other severe disease, treatment with steroids or immune-modulating treatment, pharmacological treatment of infection during the last 3 months and major cognitive dysfunction.

The ethics committee at Gothenburg University approved the study (Dnr 560–13) which was conducted in accordance with the Declaration of Helsinki. Participants gave written informed consent.

### 2.2 Anthropometry and blood pressure

Weight was measured to the nearest 0.1 kg on digital SECA 910 electronic scales (Vogel and Halke, Hamburg, Germany), with the subjects in light clothing. Body height and waist circumference were measured according to current recommendations[25].

### 2.3 Questionnaires

A questionnaire was used to collect detailed information on a variety of parameters, such as e.g., self‐reported health and smoking habits.

### 2.4 Insulin resistance

Insulin resistance was estimated with the HOmeostatic Model Assessment for Insulin Resistance (HOMA-IR) formula: (HOMA-IR) = ([insulin x capillary glucose] / 22.5).

### 2.5 Imaging and image analysis

All imaging was performed using the same CT-scanners and protocols, Siemens Somatom Definition Flash with a Stellar detector (Siemens Healthcare, Forchheim, Germany). Care Dose 4D was used for dose optimization. Cardiac image acquisition was ECG-gated, with tube voltage of 120 kV, and refmAs of 80. These images have a matrix of 512 × 512 voxels in the axial plane, with a square DFOV in the range of 170–200 mm and were reconstructed using the B35f HeartView medium CaScore algorithm, generating a slice thickness of 3 mm, with 50 % overlap between slices.

### 2.6 Analysis of CACS

Coronary artery lesions with a radiodensity of > 130 HU were classified as calcified plaques and included in the CACS according to the method described by Agatston et al[26].

### 2.7 Analysis of EATV and EATA

Non-contrast cardiac CT examinations from totally 1,945 individuals were analyzed automatically for EATV and median EATA with a fully automatic deep-learning-based model developed by our group[20]. Briefly, the model has been validated in a large series of cases from a technically identical cohort and found to generate segmentations in line with manual expert segmentations, with a Dice-score at the upper practical limit of what has been both published on manual inter-reader agreement, and what our own inter-reader Dice-score has previously been[27]. The model automatically identifies potentially flawed segmentations/analyses, which can then be manually corrected if necessary. Details on the analyses of EAT in this cohort have been described previously[28].

### 2.8 Statistics

All data was tested for normality with the Kolmogorov-Smirnov test. All data is reported with its respective median and interquartile ranges, since it was not normally distributed. The Mann-Whitney *U* test was used to test for differences in continuous variable data between groups, while the Fisher exact test was used to test for differences in categorical data. Throughout, p < 0.05 was considered to denote significance. No imputation of missing data was performed.

Three different statistical tests were used to explore a possible association between CACS > 0 and EATV or EATA.

Firstly, uni- and multivariable logistic regression analysis was used to assess the importance of EATV and EATA in relation to co-variates and confounders in explaining the presence of CACS > 0. The multivariable analyses were performed with four limited models, and one full model, including all variables significant in univariable testing or the Mann-Whitney *U* test between the CACS = 0 and CACS > 0 groups. The four limited models included the following variables: *model 1* = (age + EATV); *model 2* = (age + EATV + waist circumference + BMI); *model 3* = (age + EATV + waist circumference + BMI + systolic blood pressure + HbA1c + LDL + triglycerides); *model 4* = (age + EATV + waist circumference + BMI + systolic blood pressure + HbA1c + HOMA-IR + LDL + triglycerides + HDL + Apolipoprotein B + CRP). Linear regression modeling in the group with CACS > 0 was used in both sexes to evaluate possible associations between the level of CACS and EATV, EATA and co-variates.

Secondly, to identify the relative contribution of individual variables in explaining the binary outcome of CACS 0 or > 0, a gradient boosted tree model (GBM)[29] was applied. The overall explanatory value of the GBM-analysis can be expressed as the area under the curve (AUC). Variables contributing more than 1 % in either sex in a first screening analysis including all variables were included in the final GBM analysis.

Thirdly, the Matthews correlation coefficient (MCC)[30], [31], [32] was calculated for the prediction of the presence of calcifications (CACS > 0) based the continuous variables included in the other models. Briefly, since the individuals with calcifications are known, the ratio of true and false positives and negatives can be calculated, when ordering the individuals according to the other variables, yielding the MCC, which is a robust correlation metric in cases, where the distribution of data is not balanced between groups.

In a sensitivity analysis proportional weighting (x 4) of the data in the normal glucose tolerance (NGT) group was used to achieve closer representation of the background population.

## 3 Results

### 3.1 Data integrity

From an initial number of 1,948 individuals having valid CT-image data, 3 cases were excluded due to a lack of CACS-data. Among the included 1,945 individuals, data was missing on HOMA-IR in 19 women and 9 men, on cholesterol and lipids in 6 men and 7 women, on Apolipoprotein A1 and B in 6 men and 10 women, and occasionally (1–6 cases) on other laboratory data.

### 3.2 Demographics, anthropometric and biochemical analyses

A total of 1,064 women (54.7 %) and 881 men (45.3 %) were included, of whom 363 (34.1 %) and 573 (65 %) respectively had calcifications in the coronary arteries (CACS > 0). The median age was 58.0 years in women and 58.9 years in men. Significant differences between men and women (p < 0.05 on the Mann-Whitney *U* test) were found in all parameters except: age, LDL, Apo B, HbA1c, and EATA **(**Table 1**)**. Also, no significant difference was found in cigarette smoking (Fisher exact test). For further statistical analyses, the cohort was split by sex, and for the logistic regression and correlation analysis also according to the presence of CACS > 0.

**Table 1:** Characteristics of the cohort by sex and presence of coronary calcifications (CACS > 0). Continuous data is represented with its median and interquartile range (in brackets), while categorical data is presented as percentage of cases in each column. Significant differences between men and women are denoted with (*) and between “CACS = 0” and “CACS > 0” with ** as per the Mann-Whitney *U* test for continuous and the Fisher Exact test for categorical variables.

	**Female**, **n = 1,064** (54.7 % of all)			**Male**, **n = 881** (45.3 % of all)		
	**All (n = 1,064)**	**CACS = 0 (n = 701)**	**CACS > 0 (n = 363)**	**All (n = 881)**	**CACS = 0 (n = 308)**	**CACS > 0 (n = 573)**
Number of cases with CACS > 0 (*)	363 (34.1 %)	0 (0 %)	363 (100 %)	573 (65.0 %)	0 (0 %)	573 (100 %)
Calcification volume [mm3] (*)	0 (0–4.1)	0 (0–0)**	21.0 (3.8–66.4)**	5.7 (0–58.5)	0 (0–0)**	33.6 (6.7–125)**
Age [years]	58.0 (53.9–61.9)	57.1 (53.0–61.0)**	60.0 (56.3–63.1)**	58.9 (54.3–62.4)	56.7 (52.7–60.8)**	60.2 (55.6–62.9)**
Cigarette smoker [%]	7.9	5.7**	12.1**	9.7	7.5	10.8
Weight [kg] (*)	74.5 (66.2–83.7)	73.8 (65.9–83.1)	76.0 (67.3–85.7)	89.5 (81.4–99.8)	88.1 (80.0–96.5)**	90.4 (82.8–92.5)**
Waist circumference [cm] (*)	93 (85–102)	92 (84–101)**	94 (87–104)**	103 (95–110)	100 (93–107)**	104 (96–111)**
BMI (*)	26.7 (23.9–30.2)	26.4 (23.7–29.9)**	27.5 (24.4–31.3)**	27.6 (25.4–30.7)	26.9 (24.9–29.6)**	27.9 (25.7–31.0)**
Morbid obesity, BMI ≥ 40 [%]	1.2	1.0	1.7	1.0	0.97	1.0
Blood press., systolic [mm Hg] (*)	123 (113–135)	121 (112–133)**	125 (117–136)**	129 (120–139)	126 (117–135)**	131 (121–141)**
Blood press., diastolic [mm Hg] (*)	79 (73–86)	79 (73–85)**	80 (75–87)**	81.5 (76–89)	81 (75–87)**	82 (77–90)**
Cholesterol [mmol/l] (*)	5.7 (5.0–6.4)	5.6 (5.0–6.3)	5.8 (5.1–6.5)	5.3 (4.7–6.0)	5.2 (4.6–5.9)	5.4 (4.7–6.0)
LDL [mmol/l]	3.7 (3.0–4.3)	3.6 (3.0–4.3)	3.7 (3.1–4.4)	3.6 (3.0–4.3)	3.5 (3.0–4.1)	3.7 (3.0–4.3)
HDL [mmol/l] (*)	1.8 (1.5–2.2)	1.9 (1.6–2.2)**	1.8 (1.5–2.1)**	1.4 (1.2–1.7)	1.5 (1.2–1.8)	1.4 (1.2–1.7)
Triglycerides [mmol/l] (*)	1.0 (0.80–1.4)	1.0 (0.8–1.4)	1.1 (0.90–1.5)	1.2 (0.9–1.8)	1.1 (0.80–1.5)**	1.2 (0.9–1.9)**
Apo A1 [mmol/l] (*)	1.7 (1.6–1.9)	1.8 (1.6–1.9)	1.7 (1.5–1.9)	1.5 (1.4–1.7)	1.5 (1.4–1.7)	1.5 (1.3–1.6)
Apo B [mmol/l]	1.1 (0.90–1.3)	1.1 (0.9–2.0)**	1.1 (1.0–1.3)**	1.1 (0.9–1.3)	1.1 (0.90–1.2)**	1.1 (1.0–1.3**
HbA1c [mmol/mol]	35 (33–37)	35 (33–37)**	36 (34–38)**	35 (33–38)	35 (33–37)**	36 (33–38)**
HOMA-IR (*)	1.4 (1.0–2.1)	1.4 (1.0–2.0)	1.5 (1.0–2.3)	1.7 (1.1–2.8)	1.6 (1.0–2.5)	1.8 (1.1–2.9)
CRP [mmol/l]	1.2 (0.60–2.7)	1.1 (0.6–2.6)	1.2 (0.70–2.9)	1.1 (0.6–2.3)	0.9 (0.6–1.7)**	1.2 (0.70–2.5)**
EATV [ml] (*)	99.0 (76.0–125)	95.7 (73.8–122)**	103 (81.5–131)**	129.7 (99.6–161)	120 (92.2–150)**	135 (105–169)**
EATA [HU]	−69 (−65-(−73))	−69 (−65-(−73))	−70 (−66-(−73))	−70 (−66-(−73))	−69 (−65-(−72))	−70 (−66-(−73))

### 3.3 Relationship between EATA and EATV

**Fig. 1:**
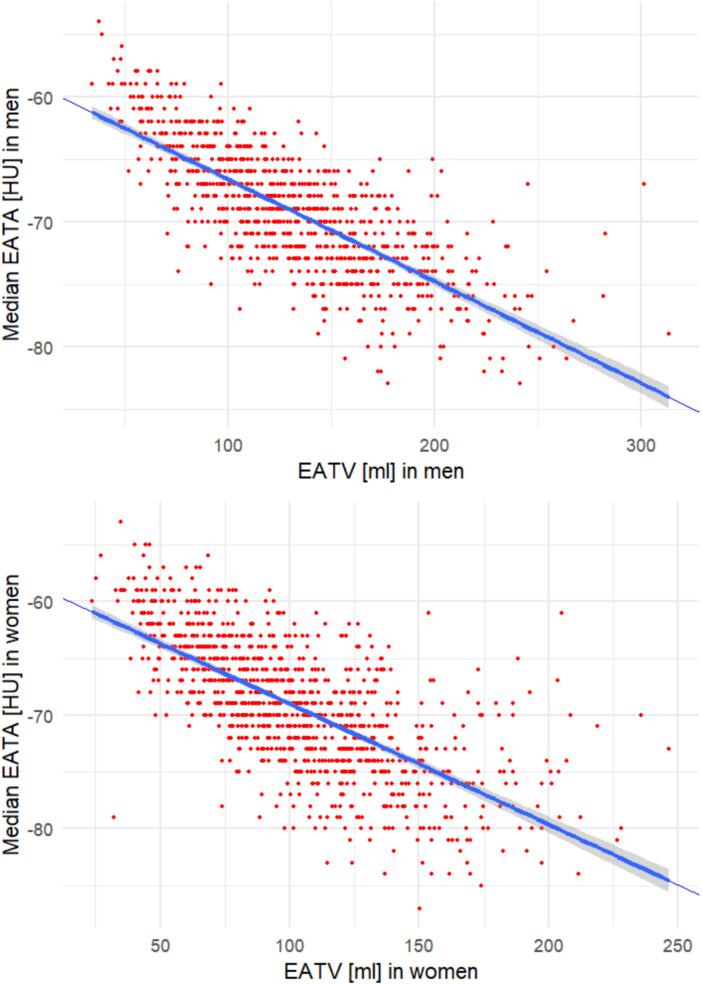
**a and 1b.** The relationship between epicardial adipose tissue attenuation (EATA) and epicardial adipose tissue volume in men and women.

There was an inverse correlation on linear regression in both sexes between EATA and EATV, with lower mean EATA among individuals with high EATV (Fig. 1**a and 1b**). In women, for each ml in increase in EATV, there was a decrease in EATA of 0.11 HU (SE 0.0034, p < 0.001). In men, for each ml in increase in EATV, there was a decrease in EATA of 0.082 HU (SE 0.0024, p < 0.001). Spearman’s correlation was −0.71 (p < 0.001) in women and −0.76 (p < 0.001) in men.

### 3.4 No association between EATV or EATA with CACS > 0 in regression analysis

In univariable logistic regression analyses EATV was associated with CACS > 0 in both men and women. However, no independent association between EATV and CACS > 0 was seen in multivariable analyses. EATA was not associated with CACS > 0 in any model. In men age, cigarette smoking and LDL-cholesterol were significantly associated with CACS > 0 in multivariable analyses. In women age and systolic blood pressure were associated with CACS > 0 **(**Table 2a**)**. Nagelkerke’s R^2^, which is an adjusted version of Cox and Snell’s R^2^, covering the full range between 0 and 1 for the coefficient of determination, was used to estimate the proportion of variance in the dependent variable associated with the various combinations of independent variables in the models. The full model, which had the highest Nagelkerke’s R^2,^ and thus the highest explanatory capability, explained 15.6 % of the variance in the presence of CACS > 0 in men and 14.8 % of the variance in the presence of CACS > 0 in women.

**Table 2a:** Significance levels of independent variables included in univariable and multivariable logistic and linear regression analysis with CACS as dependent variable. Female participants (n = 1,064) in the left and male (n = 881) in the right half of the table. The first two columns refer to the logistic regression for CACS > 0 in all (men or women), and the last two columns refer to the linear regression for CACS performed in the subgroup with CACS > 0. Statistically significant (p < 0.05) results are highlighted in bold for the multivariable analyses. “NS” denotes p > 0.05.

	**Female (n = 1,064)**												**Male (n = 881)**											
	**Univariable logistic regression, all**			**Multivariable logistic regression, all**			**Univariable linear regression if CACS > 0**			**Multivariable linear regression if CACS > 0**			**Univariable logistic regression, all**			**Multivariable logistic regression, all**			**Univariable linear regression if CACS > 0**			**Multivariable linear regression if CACS > 0**		
	Log OR	SE	p-value	Log OR	SE	p-value	Estimate	SE	p-value	Estimate	SE	p-value	Log OR	SE	p-value	Log OR	SE	p-value	Estimate	SE	p-value	Estimate	SE	p-value
Age [years]	0.115	1.54 e-02	9.5 e-14	**0.107**	**1.83 e-02**	**5.2 e-09**	3.56	2.48	NS	3.39	3.00	NS	0.110	1.64 e-02	1.4 e-11	**0.093**	**0.019**	**8.0 e-07**	13.43	3.47	1.2 e-04	10.34	3.78	**6.5 e-03**
Cigarette smoker [y/n]	0.772	0.231	8.5 e-04	**0.822**	**0.252**	**1.1 e-03**	43.08	32.21	NS	44.19	35.12	NS	0.408	0.256	NS	0.261	0.268	NS	−11.41	49.33	NS	−27.01	47.97	NS
Weight [kg]	1.29 e-02	4.76 e-03	6.7 e-03	3.95 e--03	1.36 e-02	NS	0.338	0.710	NS	0.441	2.00	NS	1.70 e-02	5.43 e-03	1.8 e-03	−2.46 e-03	1.43 e-02	NS	2.08	1.10	NS	−1.01	2.71	NS
Waist circumference [cm]	2.24 e-02	5.46 e-03	4.0 e-05	1.51 e-02	1.38 e-02	NS	0.525	0.819	NS	−0.461	1.98	NS	2.82 e-02	6.57 e-03	1.8 e-05	1.40 e-02	1.82 e-02	NS	3.66	1.35	6.7 e-03	1.13	3.52	NS
BMI [kg/m2]	5.09 e-02	1.41 e-02	3.1 e-04	3.06 e-02	3.93 e-02	NS	1.31	2.08	NS	0.580	5.63	NS	6.65 e-02	1.89 e-02	4.3 e-04	−1.29 e-02	4.97 e-02	NS	10.29	3.82	7.2 e-03	5.15	9.43	NS
Systolic BP [mm Hg]	1.65 e-02	4.08 e-03	5.2 e-05	1.07 e-02	8,26 e-03	NS	0.364	0.652	NS	0.869	1.26	NS	2.63 e-02	5.40 e-03	1.2 e-06	**2.60 e-02**	**1.02 e-02**	**1.1 e-02**	0.294	1.05	NS	0.195	1.84	NS
Diastolic BP [mm Hg]	1.99 e-02	6.99 e-03	4.3 e-03	3.73 e-03	1.44 e-02	NS	−3.90 e-02	1.12	NS	−1.64	2.16	NS	2.60 e-02	8.26 e-03	1.7 e-03	2.06 e-02	1.61 e-02	NS	−0.335	1.71	NS	−1.87	3.03	NS
LDL [mmol/l]	0.155	6.66 e-02	2.0 e-02	**−0.692**	**0.242**	**4.3 e-03**	−2.51	10.21	NS	−45.17	34.27	NS	0.160	7.76 e-02	3.9 e-02	−0.527	0.294	NS	−54.07	15.86	7.0 e-04	−103.11	55.47	NS
HDL [mmol/l]	−0.348	0.138	1.2 e-02	−0.107	0.174	NS	−11.77	20.40	NS	−2.22	26.39	NS	−0.552	0.172	1.4 e-03	−0.114	0.230	NS	−97.57	38.65	1.2 e-02	−38.99	49.07	NS
Triglycerides [mmol/l]	0.311	0.103	2.5 e-03	−0.119	0.133	NS	7.36	13.15	NS	−8.84	16.80	NS	0.372	9.89 e-02	1.7 e-04	7.94 e--02	0.129	NS	32.54	15.11	3.2 e-02	−1.55	19.43	NS
Apo B [mmol/l]	1.00	0.254	7.9 e-05	**3.29**	**0.973**	**7.3 e-04**	13.85	38.16	NS	164.93	136.07	NS	1.08	0.297	2.9 e-04	**2.80**	**1.17**	**1.7 e-02**	−119.48	59.69	4.6 e-02	238.51	217.39	NS
HbA1c [mmol/mol]	7.10 e-02	1.80 e-02	8.2 e-05	0.027	0.020	NS	−1.84	2.51	NS	−4.73	2.88	NS	4.84 e-02	1.70 e-02	4.2 e-03	1.41 e-02	1.68 e-02	NS	16.04	3.04	1.9 e-07	11.76	3.25	**3.3 e-04**
HOMA-IR	0.170	5.17 e-02	1.0 e-03	3.61 e-02	6.22 e-02	NS	6.88	6.86	NS	8.29	8.49	NS	0.110	4.87 e-02	2.4 e-02	−6.30 e-02	5.95 e-02	NS	36.92	9.45	1.0 e-04	10.16	11.52	NS
CRP [mmol/l]	1.60 e-03	1.90 e-02	NS	5.80 e-02	3.12 e-02	NS	3.91	4.67	NS	1.64	5.41	NS	9.35 e-02	3.45 e-02	6.8 e-03	3.29 e-02	2.61 e-02	NS	−1.01	2.96	NS	−2.74	2.91	NS
EATV [ml]	6.37 e-03	1.77 e-03	3.3 e-04	5.09 e-04	3.52 e-03	NS	0.389	0.274	NS	8.32 e-02	0.521	NS	8.25 e-03	1.67 e-03	7.8 e-07	7.08 e-03	3.62 e-03	NS	0.724	0.332	3.0 e-02	0.607	0.669	NS
EATA [HU]	1.94 e-02	1.15 e-02	NS	2.83 e-02	1.86 e-02	NS	−1.98	1.91	NS	−0.366	2.88	NS	4.54 e-02	1.46 e-02	1.9 e-03	4.68 e-02	2.74 e-02	NS	−3.10	3.15	NS	6.70	5.42	NS

BMI = body mass index, BP = blood pressure, LDL = low density lipoprotein, HDL = high density lipoprotein, Apo B = apolipoprotein B, HOMA-IR = homeostatic model assessment for insulin resistance, CRP = C-reactive protein, EATV = epicardial adipose tissue volume, EATA = epicardial adipose tissue attenuation

Linear regression analysis within the group with CACS > 0 showed no significant association between the severity of CACS and EATV except for EATV in model 1 (age + EATV) in men. EATV lost its significance in all the other models including the full multivariable model (Table 2a).

The various models, which tested a stepwise increase of the number of included variables, showed that the association between EATV and CACS > 0 became insignificant in both sexes after entering anthropometric data in model 2 (Table 2b).

**Table 2b:** Summary of the different combinations (“models”) of co-variates used in the multivariable logistic regression analyses and the influence on the p-value of EATV. Model 1 includes only EATV and age. Model 4 includes EATV, age, waist circumference, BMI, systolic blood pressure, HbA1c, HOMA-IR, LDL, triglycerides, HDL, Apolipoprotein B and CRP. The full model* includes all variables except Apolipoprotein A1 and cholesterol, which were not or borderline significant in univariable analysis. “NS” denotes p > 0.05.

	**Female**	**Male**
	p-value of EATV	p-value of EATV
Univariable analysis	3.3e-04	7.8e-07
Model 1	NS	2.6e-05
Model 2	NS	NS
Model 3	NS	NS
Model 4	NS	NS
Full multivariable analysis*	NS	NS

### 3.5 Gradient boosting model analysis

**Fig. 2:**
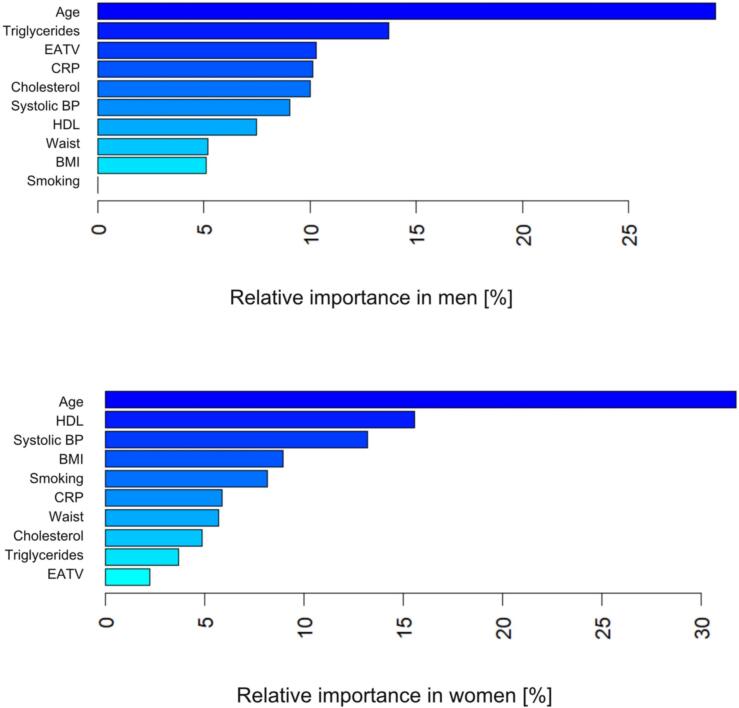
**a and 2b.** The relative contribution of different variables to the explanation of the presence of CACS > 0 in men and women in analyses performed with a gradient boosting machine (GBM).

The relative contribution of EATV to the total predictive capability of the model was 2.2 % and 10.3 % for women and men respectively in an analysis limited to the variables contributing more > 1 % in either sex in a screening analysis including all variables in Table 1. The relative contribution of EATA was low (< 1 %) in both women and men, and EATA was not included in the final analysis. Age showed the highest relative importance in both men and women in the final model **(**Fig. 2**a and 2b,** Table 3**)**. The AUC in women was 0.71 and 0.70 in men, which translates to an intermediate accuracy of prediction.

**Table 3:** Results of the Gradient Boosted Model (GBM) analysis, where the relative importance of the various variables in explaining the binary classification outcome of CACS 0 or CACS > 0 are shown (in percent).

	**Female**	**Male**
Age	31.7	29.1
EATV	2.2	10.3
Cigarette smoking	8.1	<1
Waist	5.7	5.2
BMI	9.0	5.1
Systolic BP	13.2	9.0
Cholesterol	4.9	10.0
HDL	15.6	7.5
Triglycerides	3.7	13.7
CRP	5.9	10.1

### 3.6 Correlation analysis

Of the analyzed continuous variables, none showed a strong correlation to the presence of coronary artery calcifications (CACS > 0), as expressed by the Matthews correlation coefficient (MCC). Age showed the relatively strongest correlation, with an MCC of 0.184 in men and 0.180 in women, while EATV and EATA had MCC close to 0, indicating very weak correlations **(**Table 4**)**.

**Table 4:** The Matthews correlation coefficient (MCC) for relevant continuous variables in relation to the presence of CACS > 0. An MCC of 1 denotes perfect correlation, −1 denotes perfect inverse correlation, and 0 denotes no correlation at all.

	**Female**	**Male**
Age	0.180	0.184
Weight	0.111	0.139
Waist	0.138	0.130
BMI	0.137	0.108
Systolic BP	0.0637	0.0675
Diastolic BP	0.0893	0.0616
Cholesterol	0.0666	0.0450
LDL	0.0901	0.106
HDL	0.108	0.0829
Triglycerides	0.130	0.124
Apo A1	0.138	0.0484
Apo B	0.112	0.136
HbA1c	0.08721	0.0846
HOMA-IR	0.114	0.0991
CRP	0.127	0.0965
EATV	0.0718	−0.0624
EATA	0.0917	0.0941

### 3.7 Sensitivity analyses

Analyses were repeated using proportional weighting of the data in the normal glucose tolerance group (NGT) to emulate a population more representative of the background population. Results remained very similar and the GBM-analysis showed only minor changes in the relative importance of some variables (data not shown). Age remained the most important predictor of CAC. No further exploration of EATV or EATA in relation to glucose status was performed, since this has been previously published in the same cohort[28], with results showing higher EATV with increasing glucose metabolic derangement, but not independently of other anthropometric factors.

## 4 Discussion

We have presented radiological data on coronary artery calcifications (CAC) in 1,945 individuals in relation to EAT volume (EATV) and attenuation (EATA) and established anthropometric and cardiometabolic risk factors. The cohort was selected from a normal population, but with an oversampling of individuals with glucose disorders (pre-diabetes and newly diagnosed T2D). No contrast enhanced CT images, and therefore no data on non-calcified plaques was available. Also, no clinical outcome measures were available for analysis.

Despite our material representing a large cohort with complete EATV segmentations, which is an improvement with regards to some of the previous studies, and despite using a range of statistical models and tests, we could not identify an association between CAC and EATV or EATA which remained significant after adjusting for relevant co-variates in analyses stratified for sex. Age was the single most important factor explaining differences in CAC in both sexes. Although obesity has been linked to the presence of CAC[33], [34], [35], and is an important driver of glucose disorders[36], [37], we could not verify an independent association between obesity − measured as BMI or waist circumference − and the presence of CAC in multivariable analysis. The prevalence of morbid obesity, defined as BMI ≥ 40 (type III obesity), was comparably low[38] in our cohort (around 1 %), and not significantly different across groups. Pre-diabetes and the metabolic syndrome have both been associated with increased EATV and CAC[39], [40], [41]. We had data on the various pre-diabetic stages, which has been previously explored in relation to EATV and EATA[28], and HOMA-IR as a continuous variable reflecting the severity of the derangement of glucose metabolism but could not verify an independent association between HOMA-IR and the presence of CAC. It is possible, that our cohort (with 52 % having normal glucose tolerance, 21 % having impaired fasting glucose, 16 % having impaired glucose tolerance and 10 % having combined glucose intolerance or type 2 diabetes) simply represents too early stages of glucose disorders for their effects to have translated into CAC beyond what other known risk factors account for.

Notably, the relative contribution of EATV to the total predictive capability of the GBM analysis for the presence of CACS > 0 was higher in men (10.3 % vs. 2.2 % in women) in our material, despite all other statistical models failing to detect any significant associations. This would be in line with previously reported differences in sex between the factors influencing the process of atherosclerosis, visible also in the different prevalence and symptomatology reported in clinical studies[42], [43]. What is not clear from our results is, however, to what extent this would be an age-dependent issue among women, who generally tend to be older when they develop their atherosclerosis and CAC. It is conceivable, that a similar relative contribution would have been seen in women, had they been older, and not of the same age as the men.

Moreover, EATA, which has been advocated as a more sensitive metric in estimating the risk of atherosclerotic events [16], [44] was not significantly associated with CACS in any of our analyses. This can be at least partly explained by a strong inverse correlation between EATA and EATV seen in both sexes. This notwithstanding, local changes in EATA reflecting the balance of lipid content, inflammatory cells and fibrotic tissue, might be overlooked, and there is no clear consensus on whether high or low attenuation at a total volume level signals overall risk of coronary atherosclerotic events[45], [46], [47] This of course raises questions to how reliable EATA measurements in total EAT volumes are. Plausibly, measuring EATA in the complete volume is not a valid method for the assessment of localized processes around the coronary arteries, of which inflammation induced by, e.g., vulnerable or unstable plaques would be one prominent example. In this regard, measuring the EATA adjacent to the coronary arteries has proven more successful, and should probably be the focus of future research[48], [49], [50], although currently limited to contrast enhanced CT images, why it is of little practical relevance to our cohort.

The relationship between CAC and EATV shows some divergence in the literature. In a *meta*-analysis of 18 studies comprising a total of more than 21,000 individuals, Mancio et al., summarizing most of the work in the field until 2016, found that CAC was significantly associated with increased EATV in studies not adjusting for covariates, while the association was weakened to borderline significance in studies adjusting for covariates[51]. In the same *meta*-analysis EATV was however significantly associated with coronary artery stenosis, myocardial ischemia and major adverse cardiovascular events (MACE) in both unadjusted and adjusted analyses.

Significant associations between CAC and EATV have been shown in the Framingham and MESA studies[18], [52]. Rosito et al. investigated a cohort of 1,155 individuals free from known cardiovascular disease belonging to the original Framingham cohort and found that the presence of CAC was significantly associated with total EATV[18]. Their measured EATV was very close to our results, and they adjusted for similar anthropometric and laboratory co-variates, however defined the presence of CAC in relation to the 90th percentile of a healthy normal sample. Also, their cohort represented an older population, with a mean age of 63 compared to our median of 58 and 59 for women and men respectively. Ding et al[52]. found a positive association between CAC and pericardial adipose tissue in the Multi-Ethnic Study of Atherosclerosis (MESA). In a later publication with expanded analyses from MESA on more than 6,000 individuals, McClain et al. found a weaker but still significant association in adjusted analyses[53]. It is however important to note, that they did not measure total EATV, but rather a limited volume at the level of the proximal left coronary artery, also including some extra-pericardial adipose tissue.

The relationship between EATV and cardiovascular adverse events is more consistent in the literature. In a systematic review of 29 studies comprising almost 20,000 individuals, Chong et al. found a consistent association between EATV and outcomes such as cardiac death, myocardial infarction, coronary revascularization, and atrial fibrillation[54].

The apparent discrepancy, both in our results and much of the literature, from a lack of association between EATV and CAC and a more consistent association between EATV and cardiovascular events is difficult to explain. While the pathophysiology of atherosclerosis and the formation of calcifications is reasonably well described [55], [56], [57], [58], we know very little about time related changes in EATV and its potential to induce inflammation. Atherosclerosis seems driven by inflammation [59], [60], [61], and calcification likely is a late event in this process. It may be that EAT mainly affects the early phases of atherosclerosis and not the stages when macroscopic calcifications are formed, and that EATV expansion precedes the formation of CAC. To what extent this is conceivable, is not known, although there is some evidence, that EATV shows a natural, background increase with age[62]. The relatively young age in our cohort, a point especially valid for women in the context of atherosclerosis, would reduce the chances of linking EATV expansion to CAC. Future studies using large datasets, preferably with serial measurements of EATV, would be needed to resolve this issue. In addition to a longitudinal approach, simultaneous analyses of EATV and EATA in both CTA and non-contrast CT from the same individuals, acquired at the same time points, allowing for geographically differentiated measurements in the EAT, would increase our understanding of how EATA relates to CAC.

### 4.1 Limitations and strengths

Although the current study is one of the largest to date, the number of women with radiological coronary atherosclerosis amounts to merely 363 individuals, compared to 573 among men. It can be argued that a more balanced population with regards to the presence of atherosclerosis might have improved the reliability of the findings, but it would have carried the necessity to significantly increase the age limits for inclusion of women, or to actively select women with atherosclerosis.

A relative overrepresentation of individuals with pre-diabetes and/or previously undiagnosed T2D in the cohort might raise some questions with regards to the generalizability of our results to the general population of the same age interval. It can be argued that an enrichment for pre-diabetic individuals would have increased the likelihood of finding an association between EAT and atherosclerosis, since impaired glucose metabolism is a prominent risk factor for the development of the latter, as well as coupled to increases in EAT volume. Repeated analyses using proportional weighting of the data were performed to investigate the possible net effects on the results from the oversampling of glucose disorders. In these analyses the results of the Gradient Boosting Model showed minor changes in the relative importance of some variables, but age remained the most important predictor of CAC. Also, we found no more than a weak association between CAC and HbA1c-levels, and only among male participants. Consequently, it seems that CAC in a cohort with at least this age span is only weakly associated with impaired glucose metabolism, and that the association doesn’t translate into significant association with EATV or EATA.

The imaging data available for the study comprise only non-contrast CT used for calculating the CACS. This practically limits the information available on EAT to total EATV and EATA pooled from the entire volume. If contrast enhanced images would have been available, a more differentiated approach would have been possible, with measurements closer to the coronary arteries, conceivably yielding other results, including the possible identification of non-calcified plaques. However, it should be noted that non-contrast images are easier to obtain, with virtually no contraindications, as opposed to CTA, and will probably, still for years to come, offer superior cost-benefit in a population screening setting. Therefore, the relevance of data on non-contrast CT from the background population, should be high.

Lastly, all EAT analyses were performed automatically by our previously developed software model[20], [28], with manual review and correction only in cases automatically flagged as potentially flawed. Any automatic analysis will always raise questions to its reliability, however, during our extensive testing, performance metrics have been consistently in line with manual expert measurements, and the median EATV and EATA are close to the mean or median values based on manual measurements reported in the literature.

## 5 Conclusions

In a cohort representing a cross-section of the Swedish population aged 50–64 years, but with a selective oversampling of individuals with pre-diabetes and early T2D, we found no significant association between EATV or EATA and coronary artery calcifications when adjusting for co-variates. This supports the overall trend in reporting from previous literature, that increased EATV is not independently associated with CAC. Importantly, this finding does not exclude a role of EAT in the development of non-calcified coronary atherosclerosis and an association with later coronary atherosclerotic events. Moreover, we analyzed complete EAT volumes, and our results consequently do not invalidate EATA studied at a local level adjacent to the coronary arteries as a potential predictor of coronary atherosclerotic disease.

## Funding sources

Work by David Molnar was supported by the grants from the Swedish state under the agreement between the Swedish government and the county councils, the ALF-agreement (ALFGBG-936207), and by grants from the Gothenburg Medical Society (GLS-972629, GLS-972862, GLS-985373, GLS-985448). Work by Göran Bergström was supported by 10.13039/100018296Forte, Sweden (17-01964), the 10.13039/100004411Heart and Lung foundation, Sweden (20180324), the 10.13039/501100001862Swedish Research Council (2019-01140), LUA/ALF: ALFGBG-718851, the Knut and Alice Wallenberg Foundation, the Swedish Research Council and VINNOVA (Sweden’s innovation agency). Work by Fredrik Bäckhed (FB) was supported by 10.13039/501100002706AFA insurances, Sweden, Knut and Alice Wallenberg Foundation, Sweden (2017.0026), the 10.13039/501100006630Heart and Lung Foundation, Sweden (20210366) and Grants from the Swedish state under the agreement between the Swedish government and the county councils, the ALF-agreement (ALFGBG-718101). FB is Wallenberg Scholar and Torsten Soderberg Professor in Medicine.

## CRediT authorship contribution statement

**David Molnar:** Writing – review & editing, Writing – original draft, Software, Project administration, Methodology, Funding acquisition, Formal analysis, Data curation, Conceptualization. **Elias Björnson:** Writing – review & editing, Software, Formal analysis, Data curation. **Ola Hjelmgren:** Writing – review & editing, Software. **Martin Adiels:** Writing – review & editing, Formal analysis, Data curation. **Fredrik Bäckhed:** Writing – review & editing, Resources, Project administration. **Göran Bergström:** Writing – review & editing, Supervision, Resources, Project administration, Funding acquisition, Formal analysis, Conceptualization.

## Declaration of competing interest

The authors declare the following financial interests/personal relationships which may be considered as potential competing interests: David Molnar reports financial support was provided by Göteborg Medical Society. David Molnar reports financial support was provided by Region Vastra Gotaland. If there are other authors, they declare that they have no known competing financial interests or personal relationships that could have appeared to influence the work reported in this paper.
